# Are Hibernators Toast? Global Climate Change and Prolonged Seasonal Hibernation

**DOI:** 10.1111/gcb.70659

**Published:** 2025-12-30

**Authors:** Kathrin H. Dausmann, Christine Elizabeth Cooper

**Affiliations:** ^1^ Functional Ecology Universität Hamburg Hamburg Germany; ^2^ School of Molecular and Life Sciences Curtin University Perth Western Australia Australia; ^3^ School of Biological Sciences University of Western Australia Crawley Western Australia Australia

**Keywords:** climate change, energy expenditure, hibernation, phenological mismatch, phenotypic plasticity, thermoregulation, torpor, water balance

## Abstract

This review examines the multifaceted implications of global climate change on mammalian hibernators, emphasizing physiological, ecological and phenological impacts. While high‐latitude habitats are experiencing faster overall warming, tropical and southern hemisphere regions face more unpredictable and variable climate alterations. Increasing temperature can directly affect hibernators by elevating hibernacula temperatures, shortening torpor bouts, increasing arousal frequency, and depleting energy reserves crucial for survival and reproductive success. Conversely, cold anomalies due to climate change may cause disruptive late‐season cold snaps, affecting post‐hibernation recovery and reproduction. The phenological timing of hibernation, emergence and reproduction is becoming increasingly decoupled from environmental cues, creating potential mismatches that threaten fitness and survival. Habitat modifications, including urbanisation, further modify microclimates, introducing new risks and opportunities influencing hibernation behaviour, resource availability and susceptibility to disturbances and diseases. Despite anticipated physiological resilience owing to broad thermal tolerances, many hibernating species already inhabit extreme environments and operate near their physiological limits, thus are even more at risk through ecological disruptions as climate variability intensifies. Ultimately, the capacity for adaptive phenotypic plasticity combined with ecological resilience will determine species' future persistence, with high‐latitude species potentially more vulnerable to ecological disruptions like habitat loss, predation and disrupted food webs, while tropical species face greater physiological risk.

## Graphical abstract

This review examines how global climate change impacts mammalian hibernators, emphasizing physiological, ecological and phenological aspects. Many hibernating species already inhabit extreme environments, thus are at risk as climate variability intensifies. Ultimately, the capacity for adaptive phenotypic plasticity combined with ecological resilience will determine species' survival. Species hibernating in colder climates are potentially more vulnerable to ecological disruptions like habitat loss, predation and disrupted food webs, while tropical species face greater physiological risk as their hibernation conditions are already closer to physiological maximal limits.

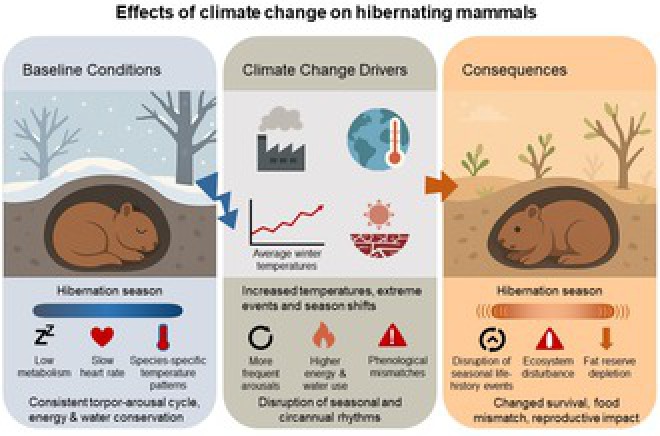

## 1 How Global Climate Change Affects the Environmental Conditions That Impact Hibernators

Climate change is a major factor contributing to global biodiversity loss (Pörtner et al. 2021). Its impact on species and ecosystems means that understanding how climate change affects species' functions is critical for wildlife conservation and management (Paniw et al. 2021). Animals' thermal physiology is key to their ability to maintain fitness in the face of climate change, by adjusting to changes in the supply of both energy and water (Fuller et al. 2020; Scopes et al. 2024). Global surface temperature over land has increased by 1.6°C from 2011 to 2020 compared to 1850–1900, and the rate of increase is escalating. Depending on future scenarios, global surface temperatures might be 4°C higher by the end of the century (IPCC 2023).

**FIGURE 1:**
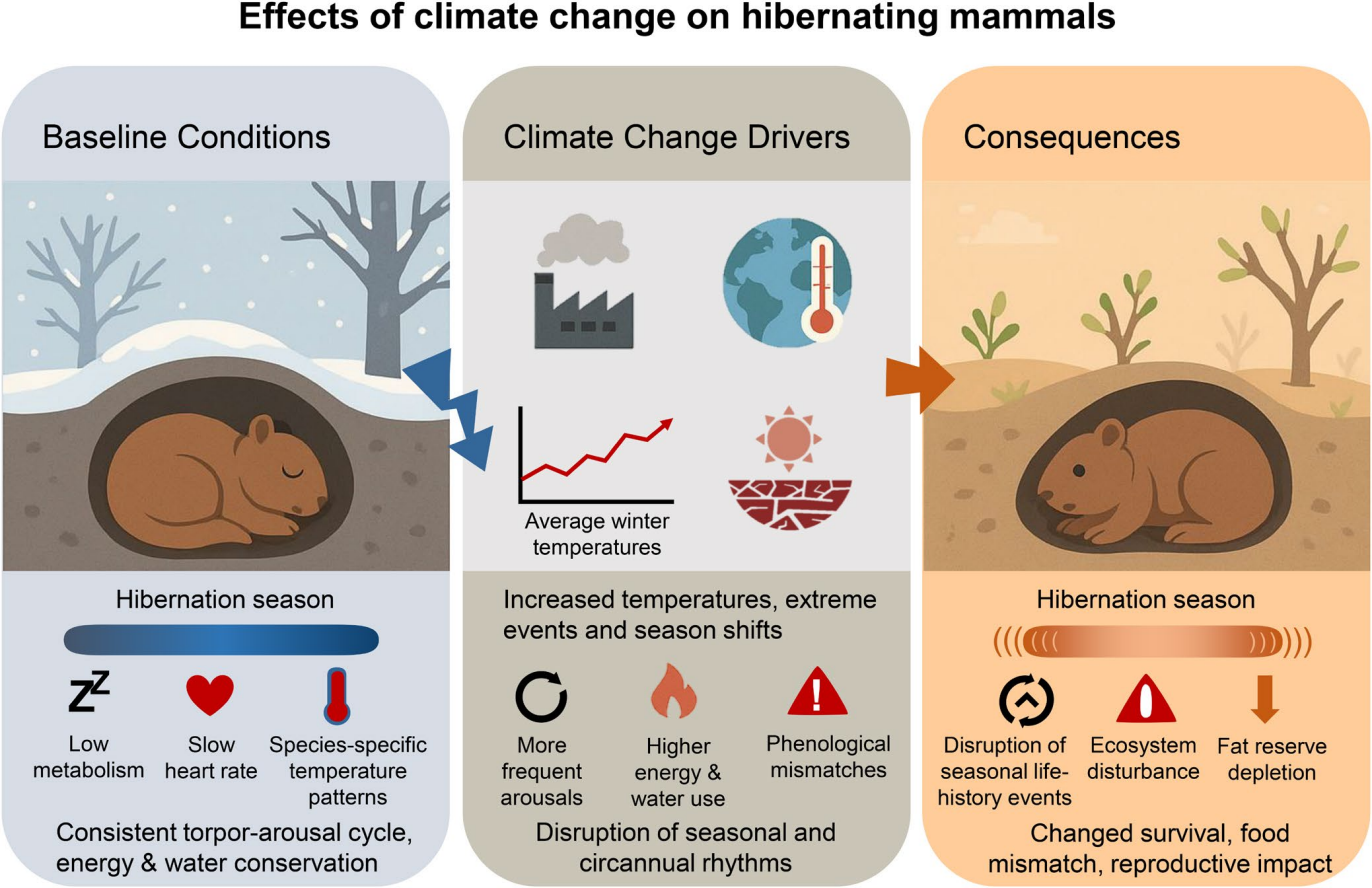
Schematic representation of the effects of climate change on hibernating mammals.

An increase in the average surface temperature is not the only impact of climate change; the global hydrological cycle is also impacted. Subtropical and mid‐latitude dry regions are becoming drier, while higher latitudes, wet mid‐latitude regions and equatorial Pacific areas are becoming wetter (Walker et al. 2019). Perhaps most critically, climate change is shifting seasons and also increasing the duration, frequency and severity of extreme environmental events such as drought, heatwaves, fires, flood and storms (Figure 1; Rahmstorf and Coumou 2011; Rummukainen 2012; IPCC 2023). These extreme events, superimposed over a generally warming climate, must be considered when predicting responses of fauna and can cause environmental bottlenecks with devastating effects on populations and even entire species (McKechnie et al. 2012; Fey et al. 2015; Morán‐Ordóñez et al. 2017; Ruthrof et al. 2018).

Although the consequences of climate change are an overall increase in global temperature and aridity, these effects are not uniform. Higher latitudes are warming faster than lower latitudes, and there is a greater absolute increase in winter compared to summer temperatures (Cohen et al. 2014; Walker et al. 2019). Northern temperate and arctic habitats are becoming warmer overall, with rising winter temperatures, more rain, less snow and a shorter duration of snowpack (Dore 2005; Rummukainen 2012; Gergel et al. 2017). Changing atmospheric circulation patterns is also making winters in some temperate and arctic regions more unpredictable (Stendel et al. 2021). In equatorial regions, warming is less and slower than observed at high northern latitudes. Temperature and especially rainfall have become more variable in the tropics, with extreme fluctuations in rainfall related to the El Niño‐Southern Oscillation (ENSO). Predictions indicate further warming with drier dry seasons and wetter wet seasons (Dore 2005; Trewin 2014). Many arid regions, such as central Australia, northern and southern Africa and the west coast of South America, will become even hotter and drier (Collier et al. 2008; Nunez et al. 2009; Head et al. 2014).

The consequences of changing temperature and hygric regimes influence all aspects of an animal's biology, as they impact energy, water and thermal balance. Temperature is one of the most pervasive variables affecting biological processes, while animals are comprised mostly of water, which is critical for animal function (Haynie 2001; Brown et al. 2004; Withers et al. 2016). Species from historically stable climates may be most sensitive to climate change, as they have not adapted to changing conditions and might have lost the regulatory genes needed for mechanisms to respond (Somero 2010). Indeed, biodiversity in tropical ecosystems is predicted to be most at risk from global climate change due to species' narrow environmental tolerances (Deutsch et al. 2008). Survival will depend on whether evolutionary adaptive processes can keep up with climatic and ecological changes or if phenotypic plasticity may mitigate some of these challenges. These responses differ widely among species, thereby establishing species' relative degrees of flexibility and ultimately sensitivity to climate change. Species capable of periods of dormancy may be particularly well suited to withstand the impacts of climate change, but some could be vulnerable due to their rigid, finely tuned life cycles and concentration in habitats experiencing some of the greatest environmental impacts. Here, we explore the potential impacts of climate change on the most well studied group of organisms undergoing periodic dormancy, the mammalian hibernators.

## 2 Prolonged Seasonal Hibernation

Environmental conditions such as extreme cold, heat, aridity or lack of resources such as food and water can pose challenges to the survival of organisms, especially for endothermic animals such as birds and mammals, which typically maintain a relatively high and stable body temperature (*T*_b_) at considerable energetic cost (Withers et al. 2016; Geiser 2020). However, some species show a remarkable ability to endure extreme environmental conditions. For example, a combination of increased thermogenesis and insulation, regional heterothermy, changes in body mass and behavioural responses allow many mammals, even small species such as voles and shrews, to remain active during winters characterised by temperatures < −30°C (Withers et al. 2016). An alternative approach to surviving harsh conditions is to avoid unfavourable environments. Migration to areas with more equable climates or regions with greater resource availability is one avoidance strategy used by endotherms (Harris et al. 2018; Robinson et al. 2009; Avgar et al. 2014). Another avoidance strategy is to become inactive in situ and ‘wait out’ the period of adverse conditions (Geiser 2021). A diverse array of animals and plants become dormant, remaining inactive and reducing their energy expenditure (Wilsterman et al. 2020). These periods of dormancy can occur from periods of < 1 day to a year or longer, and may be associated with only minor changes in thermoregulation that have an associated effect on metabolism via the Q_10_ effect, to the extreme biochemical effects of anhydrobiosis, osmobiosis, cryobios or anoxybiosis (Cooper and Withers 2024).

Here, we will discuss how one avoidance strategy, commonly known as seasonal hibernation, allows small endothermic animals to reside year‐round in highly seasonal climates (Turbill et al. 2011), and how climate change may impact them. However, it is imperative to first define what we mean by the term hibernation, because an array of terminology used to describe patterns of heterothermy and associated dormancy for endotherms (and other animals) has persisted over the last ~170 years of research into this phenomenon (Lyman 1948; Withers and Cooper 2010). Torpor for endothermic birds and mammals describes a period of controlled heterothermia, where active regulation of *T*_b_ is suspended until a new, generally lower, set‐point is reached, accompanied by a reduction in metabolic rate and evaporative water loss (Figure 1; Geiser 2021). Torpor can be differentiated from pathological hyperthermia by the ability to arouse to normothermia with endogenous metabolic heat production, but the degree of change in *T*_b_ and/or metabolic rate required to characterize torpor is contentious and varied (e.g., Barclay et al. 2001; Willis 2007; Withers and Cooper 2010). Lyman (1948) first used the term ‘deep hibernation’ to describe a pattern of deep multi‐day torpor (*T*_b_ ~ 3°C for several weeks) for golden hamsters (*Mesocricetus auratus*), and Lyman and Chatfield (1955) define ‘to hibernate’ as ‘to pass the winter in close quarters in a torpid or lethargic state’. However, this definition is not precise or mechanistic, as it includes species that are simply less active in winter than they are in summer. More recently, the term ‘hibernation’ has been expanded to include any bouts of multi‐day torpor, separating endothermic torpor into daily (< 24‐h) torpor and hibernation (torpor > 24 h), which encompasses both facultative multi‐day torpor and obligate seasonal hibernation (Geiser and Ruf 1995; Ruf and Geiser 2015). This confusing array of terms likely arises from attempts to classify the various forms of heterothermia observed for endotherms into discrete categories, when this phenomenon may be more realistically considered a continuum from strict homeothermy to extreme seasonal hibernation (Withers and Cooper 2010; Boyles et al. 2013; van Breukelen and Martin 2015).

**FIGURE 2:**
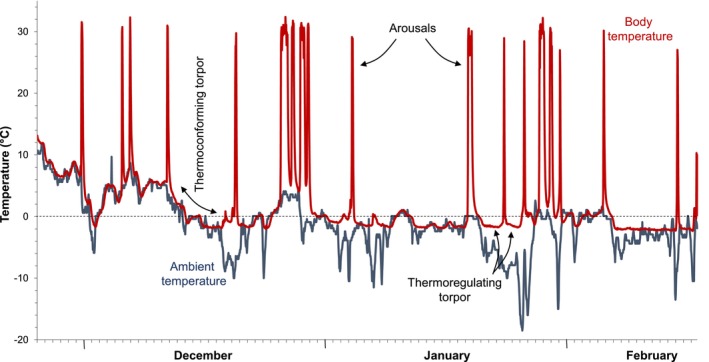
Body temperature (black line) profile of a free‐ranging hazel dormouse ( *Muscardinus avellanarius* ) undergoing prolonged seasonal hibernation for 3 months, exposed to natural weather and photoperiod near Neumünster, Germany. Ambient temperature is shown in grey. Periods when dormice thermoconform to ambient temperature, thermoregulate to maintain body temperature above ambient and undergo periodic arousals to normothermia are indicated (data from Pretzlaff et al. 2021; Dataset at UHHFDM; version 1; http://doi.org/10.25592/uhhfdm.18164).

According to the glossary of terms for thermal physiology (IUPS Thermal Commission 2003), hibernation is ‘the state of winter (…) lethargy with a reduction in *T*_b_ and metabolism of some animals that are homeothermic temperature regulators when active. (L. Hibernare—to pass the winter)’, as opposed to aestivation, which is effectively the same but associated with response to the heat and aridity of summer (e.g., cactus mouse, *Peromyscus eremicus* ; Macmillen 1965). This distinction stems from early work on hibernation for high‐latitude northern hemisphere species, but more recent work on heterothermic species at lower latitudes, which experience dry and relatively warm winters (Dausmann et al. 2004, 2020; Stawski et al. 2009; Kobbe et al. 2011; Levesque et al. 2014; Reher and Dausmann 2021) blurs this distinction. Hibernators are typically more flexible in their employment, timing and patterns of hibernation than ‘traditional’ studies on the northern high‐latitude species would have us believe (Crawford et al. 2025). Consequently, to address the terminological inconsistency that plagues this discipline and incorporate the growing body of work that documents the breadth of heterothermic responses by endothermic species, we propose the following definitions. Daily torpor: a state of reduced metabolic rate and *T*_b_ accompanied by inactivity for a period of < 24 h that is used opportunistically. Between torpor bouts, animals resume their ‘normal’ activity and there is no apparent physiological preparation. Multi‐day torpor: a state of reduced metabolic rate and *T*_b_ accompanied by inactivity for a period of > 24 h that is used opportunistically. Between torpor bouts, animals resume their ‘normal’ activity and there is no apparent physiological preparation. Seasonal prolonged torpor: a long period (weeks to months) of inactivity during which time animals enter successive bouts of deep torpor interspersed with interbout arousals during which animals ‘update’ physiologically, but usually remain in their nesting site and refrain from most other activities (Figure 2). Seasonal prolonged torpor occurs on a seasonal cycle and is characterised by physiological preparation such as fat storage and/or food caching and reduction of digestive and reproductive organs (Giroud et al. 2021; Gao et al. 2024).

Here, we will use the term hibernation to refer to those endothermic species that undertake seasonal prolonged torpor. Since most seasonally hibernating endotherms are mammalian, we will focus our review on this taxonomic group. There is only one bird species, the common poorwill ( *Phalaenoptilus nuttallii* ), where seasonal hibernation (inactivity for up to 45 days, with bouts of torpor interspersed by interbout arousals; Jaeger 1948; Woods et al. 2019) has been described. However, mammals from all three major mammalian groups (monotremes, marsupials and placental mammals), representing at least eight orders (Monotemata, Microbiotheria, Diprotodontia, Primates, Rodentia, Chiroptera, Afrosoricida and Erinaceomorpha) and inhabiting environments as diverse as the arctic tundra, Arabian deserts and Malagasy tropical forests use seasonal prolonged torpor (Withers et al. 2016; Nowack et al. 2020; Geiser 2021). Research on hibernators is highly biassed towards small mammals in northern hemisphere ecosystems. In this review, we discuss the consequences of climate change on hibernators globally, including the lesser acknowledged southern hemisphere and tropical species, which often show greater variability in their patterns of hibernation (Dausmann et al. 2004; Nowack et al. 2023).

## 3 Consequences of Warmer Temperatures for Seasonal Hibernators

Temperatures above an individual's zone of resistance can cause immediate heat‐related mortality, but even at lower temperatures within the zone of tolerance, there may be sub‐lethal consequences that reduce overall fitness (Somero 2010). Species differ in their ability to adjust their thermal tolerances in response to changing environmental conditions. Hibernating species most at risk of direct thermal impacts of climate change are those currently living close to their upper thermal tolerance limits, which are typically tropical and arid habitat species that already need to avoid these periods of high temperature and aridity by hibernating (Bartholomew and Hudson 1960; Nowack et al. 2020). These species are going to be exposed to even more extreme conditions of heat and aridity and temporal shifts in seasons. For hibernators in temperate and arctic climates, average and even extreme temperatures experienced due to global warming are less likely to be a direct thermal threat, but may have energetic and ecological consequences.

Most knowledge about seasonal hibernation under warm conditions is from Madagascar. Three groups of Malagasy animals enter seasonal prolonged torpor during the dry season: lemurs (Cheirogaleidae), tenrecs (Tenrecidae) and bats (Hipposideridae, Rhinonycteridae; Dausmann et al. 2009; Kobbe and Dausmann 2009; Lovegrove, Canale, et al. 2014; Treat et al. 2018; Reher et al. 2022). These species are more physiologically plastic while hibernating than their holarctic counterparts, reflecting their variable environments (Dausmann et al. 2022). Ambient temperature (*T*_a_) and consequently *T*_b_ is often above the torpor set‐point, so they usually thermoconform during hibernation (Dausmann et al. 2004, 2009; Kobbe and Dausmann 2009; Lovegrove, Canale, et al. 2014; Treat et al. 2018; Reher et al. 2022). Consequently, *T*_b_ depends on the climatic conditions of their habitat and the insulative properties of their hibernacula; *T*_b_ during hibernation can remain constant or fluctuate with *T*_a_. However, warm‐climate hibernation is not restricted to Madagascar. Many species of Australian tropical and arid‐habitat bats also hibernate at relatively high *T*_a_ (Geiser et al. 2020), and North American ground squirrels hibernate under conditions of heat and aridity. Mohave ground squirrels (*Xerospermophilus mohavensis*) can be inactive for 7 months as an adaptation to restricted food and water, with *T*_b_ ≥ 27°C and low humidity (Bartholomew and Hudson 1960). Richardson's ground squirrels ( *Urocitellus richardsonii* ) can enter hibernation at *T*_a_ > 20°C (Michener 1992).

In warm climates, warm hibernacula diminish the energetic benefits of hibernation compared to cold microclimates (Dausmann et al. 2009; Walsberg 2000). However, thermoconforming, combined with the absolute energy and water savings of inactivity, is beneficial for surviving seasonal aridity and food scarcity. Periodic passive rewarming also eliminates the need for energetically expensive endothermic arousals (e.g., Dausmann et al. 2004; Turbill and Geiser 2008; Lovegrove, Canale, et al. 2014). A reduced *T*_b_ and low metabolic rate provide scope for heat storage and reduce metabolic heat production (Bondarenco et al. 2014). Nevertheless, animals in warm climates rely on cool periods of the diel temperature cycle to balance their energy and water budgets during hibernation; increases in daily minima will increase *T*_b_ and therefore energy and water requirements. It is also unknown whether these species can actively defend *T*_b_ during hibernation to stay below a lethal threshold if *T*_a_ exceeds their upper critical limit. Hibernacula are usually enclosed, protected spaces, limiting radiative heat loss to the sky (Pacheco‐Fuentes et al. 2022), but there is some potential for radiative, convective and especially conductive heat exchange with cool surfaces within the hibernaculum, such as the inside of tree hollows or even cool, shaded ground (Briscoe et al. 2014; Chen‐Kraus et al. 2023; Cooper and Withers 2023). However, once environmental temperature exceeds *T*_b_, the only avenue for heat loss is evaporation. It is unlikely that warm‐climate hibernators will have access to the water required to sustain augmented evaporative heat loss, considering the winter hibernation period typically coincides with the dry season and torpor at high temperatures results in a less favourable water economy (Figure 1; Cooper et al. 2005; Withers et al. 2012). Consequently, warm‐climate species that use seasonal prolonged torpor may be at particular physiological risk of the direct impacts of climate change‐associated increases in temperature and aridity.

For hibernators in general, warming winter temperatures are altering hibernation duration (Figure 1). Although photoperiod‐entrained intrinsic circannual clocks control the timing of seasonal prolonged torpor (Körtner and Geiser 2000), environmental variables also exert an influence. Temperature is an obvious influential variable, with prolonged autumn temperatures usually delaying immergence and rising spring temperatures causing earlier emergence (Adamík and Král 2008; Sheriff et al. 2017; Fietz et al. 2020; Goldberg and Conway 2021; Findlay‐Robinson et al. 2023; Prather et al. 2023). Yellow‐bellied marmots ( *Marmota flaviventris* ), for example, responded to warming spring temperatures over a 23‐year period by emerging 38 days earlier (Inouye et al. 2000). Snow cover also impacts the timing of hibernation. Immergence dates have generally shifted backwards and emergence dates forward, with later and less snow cover and earlier snowmelt (Sheriff et al. 2011, 2017; Goldberg and Conway 2021). Advancing the commencement of the active season extends the foraging period, resulting in increased fat reserves, improved reproductive output and increased survival of the subsequent hibernation period, particularly for juveniles, as observed for Arctic ground squirrels ( *Urocitellus parryii* ; Sheriff et al. 2011). However, Sheriff et al. (2017) reported a decline in female body condition with longer active seasons, presumably because they invested more in reproduction.

**FIGURE 3:**
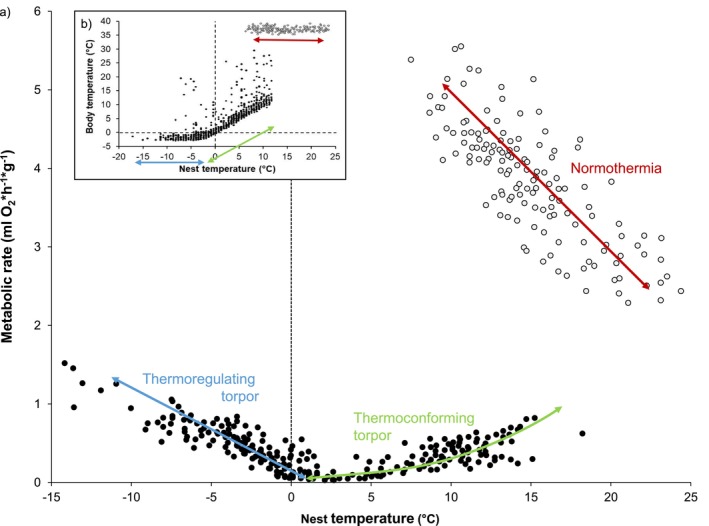
Metabolic rate (a) and body temperature (b) of hazel dormice ( *Muscardinus avellanarius* ) when normothermic (white symbols; red arrows), thermoconforming during torpor (black symbols; green arrows) and thermoregulating during torpor (black symbols; blue arrows), at a range of ambient nest temperatures (data from Pretzlaff and Dausmann 2012 and Pretzlaff et al. 2014, 2021; Dataset at UHHFDM; version 1; http://doi.org/10.25592/uhhfdm.18164).

Even cold‐climate hibernators often thermoconform during prolonged seasonal torpor bouts (Barnes and Buck 2000; Pretzlaff and Dausmann 2012; Figure 2), so *T*_b_ will be higher during hibernation with higher hibernacula temperatures, resulting in reduced energy savings due to increased metabolic rates and loss of critical fat reserves (Barnes and Buck 2000; Figure 3). In addition, torpor bout durations shorten and arousal frequencies increase at warmer temperatures, further increasing energy expenditure (Figure 1; Twente et al. 1977; Wang 1978; Geiser 2004; Geiser and Kenagy 1988; Pretzlaff and Dausmann 2012; Hoelzl et al. 2015; Geiser and Ruf 2023). Hibernators generally rely on fat or food reserves accumulated in the autumn prior to the hibernation season (Ruf and Geiser 2015), so disrupting a hibernator's energy budget can impact survival or other critical functions such as reproduction and growth and thus affect fitness (Lovegrove, Canale, et al. 2014). For many arctic species, the most advantageous soil temperature seems to be just about freezing, warm enough to forgo augmented internal thermogenesis, but cool enough to maintain extended torpor bouts (Barnes and Buck 2000; Humphries et al. 2002). Increasing *T*_a_ to 25°C (compared to 5°C and 16°C) for thirteen‐lined ground squirrels (*Ictidomys tridecemlineatus*) resulted in short daily torpor bouts instead of the usual hibernation pattern; individuals at 25°C reduced their time in torpor by > 50% (MacCannell and Staples 2021). Similarly, more frequent arousals during warm winters led to substantial increases in the energy expenditure of hazel dormice ( *Muscardinus avellanarius* ; Pretzlaff and Dausmann 2012). For eastern pygmy possums (*Cercartetus nanus*), Geiser and Ruf (2023) calculated that significantly shorter torpor bout durations when hibernating in the laboratory at *T*_a_ = 22°C would reduce the period of survival on body fat reserves to 127 days compared to 310 days when hibernating at *T*_a_ = 7°C. Reducing the hibernation duration due to increased energetic demands can have critical effects on population persistence. Northern and coastal populations of the South American marsupial Pancho's monito del monte (*Dromiciops bozinovici*) are predicted to decline under climate change scenarios ranging from optimistic to pessimistic, because there will be too few cold days for individuals to survive winter hibernation (Nespolo et al. 2024).

Warmer hibernacula temperatures can, however, be energetically advantageous. Once *T*_a_ drops below a (presumably lethal) critical set‐point, which can be as low as −2.9°C for hazel dormice (*M. avellanrius*; Pretzlaff and Dausmann 2012; Figure 2) and Arctic ground squirrels ( *U. parryii* ; Barnes 1989), hibernating mammals use endogenous metabolic heat production to regulate *T*_b_ at this, albeit relatively low, level (Geiser 2021; Figure 3). This entails substantial additional energy expenditure, which would be reduced under warming conditions that maintain the hibernacula temperature above this critical set‐point. The bat *Nyctophilus geoffroyi* hibernates in thermally variable locations under bark, where they benefit from diurnal heating by 10°C–20°C per day, greatly reducing energy expenditure during hibernation (Turbill and Geiser 2008). These energetic savings can have reproductive consequences. Warmer temperatures during winter hibernation result in heavier testes mass for male Arctic ground squirrels, although ovarian mass in females decreases (Chmura et al. 2023). Warmer winter temperatures may also allow hibernators to expand their distribution. The common noctule ( *Nyctalus noctula* ) is an insectivorous bat that breeds at northern latitudes during summer and migrates 1200–1600 km south to hibernate during winter. Over the last 30 years, the species has expanded its hibernation range northwards, presumably as a consequence of a changing climate, allowing it to forgo migration. Despite the obvious benefits of reduced migration, it remains to be seen if bats will be able to sustain their energetic requirements at these higher latitudes, with shorter photoperiod and more frequent extreme events such as late‐season cold spells (Kravchenko and Furmankiewicz 2024).

For many hibernators, the ecological effects of a warming climate may be complex. For some species, these indirect ecological effects could be of greater concern than the direct physiological impacts of changing environmental conditions. For some marmot species, global warming is promoting tree and shrub invasion of the alpine meadows they inhabit, limiting their access to palatable vegetation during the active season (Armitage 2013). Resource limitation (i.e., food and water), not temperature, is now recognized as the primary reason for seasonal prolonged hibernation (Wells et al. 2022), although some species (e.g., edible dormice, *Glis glis* ; Idaho ground squirrels and *Urocitellus brunneus* ) extend the hibernation period beyond that optimal for energetics, presumably due to other ecological benefits, such as reduced predation risk (Bieber et al. 2014; Allison et al. 2025). For sympatric species, hibernation by one species might reduce competition during resource bottlenecks; for example, lemur species within the family Cheirogaleidae (*Microcebus* ssp. and the hibernating *Cheirogaleus* ssp.) in Malagasy forests (Schwab and Ganzhorn 2004; Kamilar et al. 2016) or two ground squirrel species ( *Ammospermophilus leucurus* and the hibernating *X. mohavensis* ) in the Mohave desert (Bartholomew and Hudson 1961). Reducing the hibernation period may enhance interspecific competition, and obligate seasonal hibernation may no longer be the optimal strategy when climatic conditions allow foraging. Missed opportunity costs may disadvantage obligate seasonal hibernators compared to species that remain euthermic and active year‐round, or that use more flexible facultative single‐day or multi‐day torpor. Warmer winters in colder climates in particular may enhance winter food and water availability, eliminating the selective advantage of seasonal hibernators and opening ecosystems to other species, including competitors and predators (Figure 1; Geiser 2013; Boyles et al. 2020).

One ecological advantage of hibernation is reduced mortality from predation due to long periods of inactivity (Turbill et al. 2011; Bieber et al. 2014; Allison et al. 2025). Shorter hibernation durations, which increase the length of activity periods, or more favorable conditions that allow predators access to hibernacula, can make hibernating species vulnerable to predation, especially by introduced species (Scopes et al. 2024). For example, the mountain pygmy possum ( *Burramys parvus* ) uses seasonal prolonged torpor during the austral winter (Körtner and Geiser 1998). Warmer temperatures and less winter snow in alpine regions increase the numbers of feral cats ( *Felis catus* ) and foxes ( *Vulpes vulpes* ) that can access the possums' habitat and prey on the possums (Broome et al. 2012). In New Zealand, warmer temperatures increase beech (*Nothofagus*) seed mast events, which increase introduced rodent and in turn stoat ( *Mustela erminea* ) numbers, leading to increased predation on hibernating long‐tailed bats ( *Chalinolobus tuberculatus* ), resulting in significant population declines (Pryde et al. 2005).

Within urban landscapes, the altered microclimate (‘heat‐island’ effect of cities) and human interference can mitigate or intensify effects of climate change (Pickett et al. 2001). In addition to increased environmental temperatures, direct disturbance may also cause more frequent arousals (Speakman et al. 1991; Luo et al. 2014) and may also impact foraging efficiency during the active period. On the other hand, abundant and more stable food availability (although often nutritionally deficient; Heiss et al. 2009; Wist et al. 2022), provided nesting opportunities and artificial watering may buffer urban animals against the increasing seasonal fluctuations in resource availability and offset the negative energetic consequences (Lowry et al. 2012; Gazzard and Baker 2020).

## 4 Consequences of Cooler Temperatures for Seasonal Hibernators

The consequences of global climate change are not just restricted to an increase in mean annual temperature. As winters become more variable (Stendel et al. 2021), some northern hemisphere regions are experiencing more frequent late spring ‘freak cold snaps’ (Schmidt et al. 2024). These can disrupt resource availability for hibernators, who have just emerged and are engaged in critical post‐hibernation activities, such as reproduction and refuelling. In some locations, late‐season snowstorms reduce spring temperatures and delay snowmelt. This has delayed emergence dates for female Columbian ground squirrels ( *Urocitellus columbianus* ) by 0.47 days per year over a 20‐year period, leading to a decline in individual fitness and reduced population growth (Lane et al. 2012).

Paradoxically, some hibernators will experience colder conditions during hibernation due to climate warming, which decreases the depth and duration of snow cover (Schöner et al. 2018). Besides offering protection from predators (Goldberg and Conway 2021), snow cover has an insulative effect and helps to maintain a constant temperature within hibernacula (Young 1990; Tafani et al. 2013). Less snow means more variable, possibly lower, hibernacula temperatures, so hibernating animals are less likely to be able to passively thermoconform while maintaining *T*_b_ above freezing. This necessitates additional energy expenditure and depletes energy stores once temperatures drop below the lower critical temperature (Ortmann and Heldmaier 2000; Broome et al. 2012; Pretzlaff and Dausmann 2012). For example, the overwinter survival of mountain pygmy possums in the Australian Alps is positively related to the duration and extent of winter snow cover, which is declining with global warming (Broome et al. 2012). Likewise, Johnston et al. (2020) attributed a 74% decline in hoary marmot ( *Marmota caligata* ) abundance in the decade prior to 2016 to exposure to extremely cold and dry air in their hibernacula, leading to increased energy expenditure and more frequent arousals during winter hibernation, as a consequence of reduced insulating snow cover.

## 5 Consequences of Phenological Mismatches

Drivers of the phenology of seasonal hibernation, which involves the timing of immergence and emergence and of periodical arousals, are variable and poorly understood. Hibernation results from extension of the circadian clock, achieved by molecular inhibition of circadian cycles (Williams et al. 2014). The function of a circannual clock is also important, allowing species to maintain seasonal patterns of hibernation, arousal and reproduction even when environmental cues are unavailable, such as within sheltered hibernacula. It facilitates access to seasonal, short‐lived resources during the activity period (Körtner and Geiser 2000; Scopes et al. 2024). If an individual emerges from hibernation too early, forage conditions may be suboptimal, while emerging too late reduces the time an individual has access to food while it is most nutritious and easily digested compared to later in the summer when plants dry out and senesce (Goldberg and Conway 2021).

To maintain the entrainment of the circannual clock with seasonal environmental cycles, endocrine control of the clock is mediated by photoperiod, which causes changes in melatonin secretion prior to hibernation (Körtner and Geiser 2000; Fuller et al. 2020). Selection for species to use photoperiod, which is essentially invariant between years and has therefore been a consistent and reliable *zeitgeber* for events such as hibernation, is now challenged by asynchrony between temperature‐influenced ecological events and photoperiod. Global climate change impacts temperature but has no influence on daylength (Wingfield 2015; Walker et al. 2019). The uncoupling of seasonal events from photoperiod typically results in delayed winter conditions and advanced spring, with associated altered timing for ecologically important occurrences such as snow cover, arrival of ‘wet season’ rain, and greening and other primary production relative to day length (Donnelly et al. 2011; Walker et al. 2019). Consequently, hibernators face potential phenological mismatches between the timing of immergence and emergence and the availability of the resources needed for survival and reproduction in the active period (Scopes et al. 2024). An example of phenological mismatch for a seasonal prolonged hibernator that impacts both survival and reproduction is provided by the critically endangered mountain pygmy possum. Bogong moths (*Agrotis infusa*) are a major component of the possum's diet after its spring arousal. Moths migrate > 1000 km from their overwintering grounds to the Australian Alps, where they congregate during spring and summer, providing a protein‐rich food source for possums after arousal from, and during fattening in preparation for, hibernation. These moths are of particular importance for reproductive females and juveniles needing to fatten before their first winter (Smith and Broome 1992). Increased winter temperatures lead to early spring snowmelt, causing possums to arouse from hibernation before the arrival of the bogong moths. Foraging for alternative food then exposes the possums to increased predation risk from introduced feral predators (Broome et al. 2012).

For species undergoing seasonal prolonged torpor, advantageous physiological phenotypic plasticity may involve the flexibility to uncouple heterothermic responses from fixed environmental cues such as day length and respond opportunistically to more immediate environmental conditions (Dausmann et al. 2023). It is unclear which, if any, mammalian hibernators will have sufficiently rapid rates of adaptive evolution to match the pace of phenological shifts relative to photoperiod. To understand and predict climate change‐associated risk of phenological mismatches for populations and species, we need a greater understanding of the environmental and neuroendocrinological control mechanisms for hibernation and other seasonal events (Wingfield 2015). However, we do know that hibernation depth and duration are mediated by environmental conditions including temperature and therefore at least some species may have sufficient existing plasticity to modify the phenology of hibernation to avoid potential ecological mismatches (Scopes et al. 2024). In the case of the mountain pygmy possum, a lower‐altitude population has been discovered, which together with fossil evidence of a recent cool‐temperate rainforest distribution, varied diet, and a flexible hibernation strategy (Broome et al. 2012; Hawke et al. 2019), provides hope that this species is not necessarily an obligate alpine specialist tied to a rigid seasonal pattern of long‐term multiday torpor. Mountain pygmy possums can likely physiologically and behaviorally withstand the temperature increases associated with global warming, in part by reverting to facultative prolonged, but not strictly seasonal, torpor. However, it may be that other effects of climate change such as water availability (Cooper and Withers 2014; Bates 2017), or competition with dasyurid marsupials and rodents (Broome et al. 2012) threaten their continued persistence more than the challenges of highly seasonal long‐term torpor in a warming climate. Indeed, for many cold‐climate species, especially those at high latitudes, it is these ecological constraints, related to competition, predation and primary productivity during warmer, but still dark, winters that may pose the greatest limitations to adjusting to climate change (Angilletta et al. 2006; Geiser 2013). Many species mate shortly after emerging from hibernation, so changes in the timing of emergence may influence parturition date, with potential effects on subsequent resource availability for lactating mothers and juveniles (Dobson and Michener 1995). Fitness consequences could also arise from within‐species sex‐differentiated phenological shifts. For example, female Arctic ground squirrels are more flexible in response to climate‐change driven changes to spring temperature and snow cover than males. Over the past 25 years, females have aroused from hibernation earlier, presumably to take advantage of the resources available during an early growing season, while the date that males terminate hibernation has not changed (Chmura et al. 2023). With continued warming, these sex differences may lead to divergence of the timing of reproduction and impact breeding success for this species. For a population of Richardson's ground squirrel, females aroused from hibernation early during a particularly warm spring and became sexually receptive before many males were producing motile sperm. However, there were enough males in the population in reproductive condition that there was no impact on reproductive output (Kucheravy et al. 2021). The long‐term effect of phenological mismatch between sexes is unclear. Individual differences in the timing of male reproductive condition may provide the natural variation necessary for adaptive selection to act on, or the population could experience a genetic bottleneck as a consequence of a dramatically reduced effective population size.

## 6 Hibernators and Extreme Weather Events

The increase in the frequency, severity, and duration of extreme environmental events such as storms, floods, fires, droughts and heatwaves is perhaps a more important consequence of anthropogenic climate change for wildlife than a general increase in temperature (Figure 1; for example, Parmesan et al. 2000; Maxwell et al. 2018). These events can pose an immediate threat by impacting resource availability, imposing environmental conditions that may exceed an animal's zone of resistance or disrupting life cycles. They are also typically unpredictable and consequently require an immediate, unplanned response (Wingfield 2015). A species' flexibility with respect to hibernation may determine how extreme events impact survival and reproduction. For example, Doty et al. (2016) observed reduced torpor use by insectivorous bats ( *N. geoffroyi* ) in response to 20‐fold higher post‐fire insect abundance after a bushfire.

In situ avoidance of unfavourable environmental conditions is a major role of torpor (Geiser 2021), but the immediate avoidance of stochastic extreme events may be more relevant to those species that use facultative daily or multi‐day torpor for example, bats ( *Scotorepens greyii* , *Mormopterus* spp.) during heat waves (Bondarenco et al. 2014), bats ( *Lasiurus cinereus* ) and sugar gliders ( *Petaurus breviceps* ) during storms (Willis et al. 2006; Nowack et al. 2015), brown antechinus ( *Antechinus stuartii* ) and short‐billed echidnas ( *Tachyglossus aculeatus* ) after fire (Stawski et al. 2015; Nowack, Cooper, and Geiser 2016), than to those species which use prolonged seasonal torpor that is typically associated with prior preparation and often relies on seasonal cues such as photoperiod (Williams et al. 2014). However, even seasonal hibernation may aid survival of extreme events. If these events occur while animals are hibernating in a secure, sheltered location, they may be protected from both the extreme event and the aftermath. Indeed, the ability to hibernate in secure refugia may have enabled mammals to withstand the short and long‐term consequences of the meteorite impact that occurred at the Cretaceous‐Palaeogene boundary (Lovegrove, Lobban, and Levesque 2014).

Despite its advantages, hibernation during extreme events can be risky. The low *T*_b_ and metabolic rate associated with prolonged torpor means that individuals may have limited capacity to respond to extreme events, even if they can sense them (Nowack, Delesalle, et al. 2016). For example, Nowack, Cooper, and Geiser (2016) monitored echidnas ( *T. aculeatus* ) during a prescribed burn. Two echidnas were torpid in the same log when the burn started; one aroused, left the area and survived while the other remained torpid in the log and perished. For seasonal, prolonged torpor, the predictable availability of resources to facilitate pre‐hibernation fattening, or post‐hibernation refuelling is essential (Geiser 2013). If extreme events cause a decline in resources at these critical times, hibernators may not have the energy reserves to withstand the hibernation period or may not survive on arousal. Severe drought superimposed on a generally warming and drying climate has caused a dramatic overall decline in Bogong moth numbers in the Australian Alps (Green et al. 2021), and this together with reduced availability of the mountain pygmy possum's other primary food source, *Podicarpus* seeds, caused by increased frequency and severity of wildfire in alpine habitats, limits the overall availability of the high protein and fat diet possums need to replenish energy stores, sustain female reproductive output, and facilitate juvenile overwinter survival, compromising the ongoing health and persistence of this critically endangered marsupial (Gibson et al. 2018).

## 7 Impact of Environmental Change During the Activity Season

Overwinter survival rates are typically high for hibernating species, due to inactivity away from predators and slow ‘pace of life’ (Schaub and Vaterlaus‐Schlegel 2001; Bryant and Page 2005; Turbill et al. 2011). However, survival during hibernation and fitness‐relevant life‐history traits such as reproduction can be influenced by environmental conditions during the activity period. Most hibernators have long gestation periods with breeding precisely timed within the short active season to align high energy demands during pregnancy, lactation and weaning with climate and phenology of the habitat. This finely tuned life cycle usually allows for only one or fewer breeding events per year (Fietz and Dausmann 2006; Ruf et al. 2006), making hibernators extremely susceptible to environmental changes or disruptions (Boutin and Lane 2014).

Increasing temperature in temperate and arctic climates shifts vegetative growing seasons and other phenological events (Ruf and Geiser 2015). Increasing and more severe droughts inhibit plant growth and reproduction and, together with elevated temperature and CO_2_ levels, can change the fatty‐acid or secondary compound composition of vegetation (Lambers 1993; Beale et al. 2018; Frank 2011; Jamloki et al. 2021). This then impacts the overall availability and quality of resources or shifts the timing of their availability, affecting energy availability during hibernation in the form of fat deposits or food stores (Walsberg 2000; Geiser 2010). Changes in the quality of available resources may affect patterns and depth of hibernation. Due to changes in membrane fluidity and the functioning of trans‐membrane proteins, diets deficient in polyunsaturated fatty acids lead to less deep hibernation bouts (shorter torpor bouts with higher *T*_b_) and thus faster energy depletion (Ruf and Arnold 2008), at least in holarctic hibernators. Moreover, a common response to warmer temperature in endotherms is to voluntarily reduce food intake independent of diet‐induced thermogenesis, even when food is available and accessible, with a negative impact on growth, health and reproductive fitness (Youngentob et al. 2021).

There are numerous examples of hibernating species experiencing both positive and negative impacts of active season climate change impacts. Some populations of yellow‐bellied marmots now enter hibernation with increased body mass as a consequence of the lengthening growing season, which results in decreased mortality during hibernation (Ozgul et al. 2010). However, the overwinter survival of other populations is declining due to increased heat and aridity during the preceding summer impacting food quality (Cordes et al. 2020). Reduced survival and fecundity of Idaho ground squirrels has been attributed to changes in summer precipitation as a result of climate change (Goldberg and Conway 2021), and overwinter survivorship in golden‐mantled ground squirrels ( *Callospermophilus lateralis* ) was very low (12%) in periods of drought when they could not fatten sufficiently before hibernation (Vallance 2022). For the fat‐storing edible dormouse, higher pre‐hibernation body mass is related to later emergence from hibernation, suggesting extended hibernation is advantageous if it is energetically possible. Dormice reproduce late in the summer to synchronise with seeding of food tree species, meaning there is little to be gained from arousing earlier (Fietz et al. 2020). In other hibernating species that engage in reproduction early in the active season (e.g., little brown bats, *Myotis lucifugus* , and yellow‐bellied marmots), heavier individuals emerge first, maximising time for their offspring to grow and fatten before the next hibernation seasons (Dobson and Michener 1995; Czenze and Willis 2015; Edic et al. 2020).

## 8 Climate Change and Physiological Plasticity of Hibernation

Most predictions of species' climate change responses use biophysical distribution models based on current distribution and observed physiological traits, and relate these to predicted future patterns of climate. However, physiological traits are not necessarily fixed, with potential for adaptive genetic and phenotypic plasticity, but the degree by which plasticity will facilitate physiological and behavioral accommodation of climate change is unknown (Chown et al. 2010; Fuller et al. 2010). Since seasonal prolonged torpor is related to a long lifespan and slow generation times (i.e., hibernators are on the ‘live slow live long’ end of the ‘pace of life’; Turbill et al. 2011), genetic adaptation by hibernating mammals may be too slow to respond to the rapid changes in environmental conditions associated with anthropogenically driven climate change. Consequently, existing phenotypic plasticity is the most likely mechanism by which seasonal hibernators can respond to climate change and persist in their current environment.

The thermal biology of species throughout their geographical distributions provides an opportunity to examine plasticity in the characteristics of long‐term seasonal torpor that may reflect species' capacity to adjust to the changing environmental conditions associated with anthropogenic climate change. For example, genetic comparisons of the two monito del monte species found that the frequency of alleles associated with carbohydrate metabolism, stress responses and regulation of muscle and the nervous system relate to patterns of rainfall, temperature and elevation, consistent with local adaptation to climate (Quintero‐Galvis et al. 2024). For many species, air and/or soil temperatures and the timing of snowmelt influence hibernation duration (Lane et al. 2012; Turbill and Prior 2016; Williams et al. 2017; Constant et al. 2023; Findlay‐Robinson et al. 2023). Indeed, differences in the immergence and emergence between individuals and locations suggest a degree of plasticity in the phenology of hibernation that may allow species using seasonal prolonged torpor to respond to climate change (Williams et al. 2014). Phenological differences in hibernation for sciurid rodents are well‐recognised, with altitudinal and latitudinal patterns amongst populations (Williams et al. 2014). In some cases, activity for Arctic ground squirrel populations in relatively close proximity (e.g., 20 km apart) varies by nearly 2 weeks, presumably because of differences in the timing of snowmelt and snow cover and sufficient phenotypic plasticity to accommodate these differences (Sheriff et al. 2011). Hibernating bats ( *N. noctula* ) similarly modify characteristics of hibernation such as bout length and interbout activity intensity and duration in response to *T*_a_, and their success in colonising new, more northerly habitats has been attributed to this flexibility (Kravchenko and Furmankiewicz 2024).

Plasticity is not limited to geographically separate populations of hibernators; individual plasticity is also apparent. Free‐ranging marsupial western pygmy‐possums ( *Cercartetus concinnus* ) in a Mediterranean climate used flexible torpor patterns of including both short and prolonged torpor bouts during winter, instead of more classical hibernation, with considerable variation and a lack of synchrony between individuals (Turner et al. 2012). Under controlled laboratory conditions, intraspecific variation in the thermal biology of Belding's ground squirrels ( *Urocitellus beldingi* ) was related to male size rather than age, suggesting that even individual animals have sufficient plasticity to modify the timing of hibernation to better balance their energy budgets (French 1982). Hedgehogs ( *Erinaceus europaeus* and *Hemiechinus auritus* ) also show considerable flexibility in hibernation patterns between species, and intraspecifically between individuals related to age, sex, resource availability, location and weather (Dmi'el and Schwarz 1984; Rasmussen et al. 2019; Gazzard and Baker 2020; Crawford et al. 2025).

The short‐billed echidna ( *T. aculeatus* ) has a distribution that encompasses the entire Australian continent, with the broadest climatic distribution of any hibernator, inhabiting hot dry deserts, temperate woodlands, tropical rainforest and alpine areas (Grigg et al. 1989). As monotremes, echidnas are also a model of an advanced protoendotherm and therefore may provide information about the evolution of endothermy and patterns of torpor use (Grigg et al. 2004). Echidnas use all forms of torpor, presumably as a mechanism of ‘energetic advantage rather than energetic necessity’ over a wide range of habitats, climates and environmental conditions, in response to disturbances such as fire, even when food is available, and often commence hibernation before and terminate hibernation during the coldest part of the year to facilitate reproduction (Grigg and Beard 2000; Nowack, Cooper, and Geiser 2016). This considerable variation in echidna torpor, evaluated in context with other examples of mammalian hibernators, could be interpreted as compelling evidence of torpor states representing a facultative continuum of mild heterothermy to seasonal prolonged torpor (van Breukelen and Martin 2015), and suggests that heterothermic species have considerable capacity to modify their thermal biology to accommodate the changing ecological demands associated with global climate change.

## 9 Conclusion

How vulnerable a species is to climate change can be evaluated by their exposure (degree of climate change the organism experiences), sensitivity (tolerance, ecology and genetics), resilience (response to perturbations) and capacity for adaptation (both genetic and plastic) to these environmental changes (Williams et al. 2008; Huey et al. 2012). Species characterised by seasonal prolonged torpor are typically associated with highly seasonal climates, which occur primarily in high‐latitude or high‐altitude temperature regions, but also the wet‐dry tropics. As the effects of climate change are greatest in arctic environments, and most threatening in the tropics, hibernators are potentially exposed to the most severe effects of climate change (Geiser 2013). To date, the high‐altitude environments inhabited by many hibernators, particularly rodents, still receive reliable snow cover (Findlay‐Robinson et al. 2023), but this is likely to change as the effects of anthropogenic climate change intensify.

Perhaps surprisingly, considering the array of challenges posed for hibernators by climate change that we have highlighted here, their general sensitivity is not pronounced. A response to climate change, in terms of range contraction or shift, abundance, or changes in phenology, genetics or morphology indicating sensitivity, has been detected for 59% of North American mammal populations, with hibernation (and heterothermia) amongst the traits associated with general stability (McCain and King 2014). This observation may be in part a consequence of alpine hibernators having little to no geographical scope for movement to higher elevation or more southerly latitudes as their alpine habitat shrinks (Urban 2018; Hoffmann et al. 2019). Indeed, McCain (2019) and McCain et al. (2021) identified alpine marmots and some species of ground squirrel as amongst the North American mammals most at threat of global climate change. In general, body size and activity time were the major drivers of climate change responses, with larger mammals and those with inflexible daily activity periods most likely to be impacted, generally negatively (McCain and King 2014; McCain 2019). The relatively small size of seasonal hibernators, their ability to remain inactive for long periods, and their physical avoidance of the most extreme environmental conditions presumably buffers them from many climate‐associated threats (Geiser 2013). Studies focusing on the life‐history traits of seasonal hibernators globally found highly variable species, age, sex and population responses, with some positive effects, particularly for traits such as increased fecundity associated with higher primary productivity (Wells et al. 2022; Findlay‐Robinson et al. 2023). Moreover, hibernators generally have a higher survival rate and longer lifespan than similarly sized non‐hibernating species, presumably due to annual periods of long inactivity away from predators and other threats (Turbill et al. 2011; Ruf and Bieber 2023), and this increased lifespan can extend survival over more breeding seasons, increasing potential lifetime reproductive output (Grigg and Beard 2000).

Most hibernators typically have broad thermal tolerances, rarely experience or remain inactive at *T*_a_ close to upper critical limits, and few inhabit the stable lowland tropical habitats associated with limited acclimatory scope (Huey et al. 2012). Therefore, most hibernators are likely to have the physiological tolerance to withstand at least some extent of global warming. Nevertheless, some inhabit extreme environments, where species are already surviving at the limits of their plasticity (Fuller et al. 2010; McCain and King 2014). For these species, the consequence of climate change on climate severity may determine if there is potential physiological resistance, for example, arctic species will experience a warmer, less extreme climate, but Malagasy species hibernating to survive winter drought might not have the physiological capacity to withstand even hotter, drier conditions and a later onset of the rainy season. The concept of ‘plastic floors and concrete ceilings’, formulated for fish in a warming world (Sandblom et al. 2016), could thus also become reality for hibernators with climate change. Species hibernating in warmer climates are those likely to be at the greatest physiological risk of anthropogenic climate change, as their hibernation conditions are closer to the ceiling than the floor, and it is on these species that we should focus physiological research efforts, in particular to determine their vulnerability to the impact of increased temperature and drought and therefore susceptibility to trends of increased heat and aridity (Wells et al. 2022). Cold‐climate hibernators, however, may be more at risk of the ecological consequences of global warming than a physiological inability to withstand warmer conditions. For these species, it is important to consider the indirect effects of climatic climate change on associated species and ecological interactions (Findlay‐Robinson et al. 2023). Consequently, for cold‐climate hibernators, an ecological, rather than physiological, approach to climate change research and threat mitigation may be most fruitful.

## Author Contributions

**Kathrin H. Dausmann:** conceptualization, visualization, writing – original draft, writing – review and editing. **Christine Elizabeth Cooper:** conceptualization, visualization, writing – original draft, writing – review and editing.

## Conflicts of Interest

The authors declare no conflicts of interest.

## Data Availability

The data that support the Figures 2 and 3 of this review are openly available in UHHFDM at http://doi.org/10.25592/uhhfdm.18164.
